# Three-year audiological outcomes of the latest generation middle ear transducer (MET) implant

**DOI:** 10.1007/s00405-020-06031-6

**Published:** 2020-05-13

**Authors:** Henryk Skarżyński, Beata Dziendziel, Elżbieta Włodarczyk, Piotr H. Skarżyński

**Affiliations:** 1 Oto-Rhino-Laryngology Surgery Clinic, World Hearing Center, Institute of Physiology and Pathology of Hearing, Mokra 17, Warsaw/Kajetany, 05-830 Nadarzyn Poland; 2 Teleaudiology and Screening Department, World Hearing Center, Institute of Physiology and Pathology of Hearing, Mokra 17, Warsaw/Kajetany, 08-830 Nadarzyn Poland; 3 Heart Failure and Cardiac Rehabilitation Department, 2nd Faculty of Medicine, Medical University of Warsaw, Warsaw, Poland; 4 Institute of Sensory Organs, Warsaw/Kajetany, Poland

**Keywords:** Active middle ear implant, Middle ear transducer, Cochlear MET, Complications, Partial deafness treatment, Hearing loss

## Abstract

**Purpose:**

To evaluate the long-term audiological outcomes and safety of the latest generation of middle ear transducer (MET) among a group of Polish patients.

**Methods:**

Ten patients aged 48–72 years with bilateral sensorineural hearing loss (*n* = 8) and mixed hearing loss (*n* = 2) were included in this study. Pure tone audiometry, sound thresholds, word recognition scores in quiet and speech reception thresholds in noise were assessed. Medical and technical complication information was gathered.

**Results:**

All the patients underwent unilateral implantation with the latest generation Cochlear MET between 2014 and 2016. Mean length of follow-up was 3.7 years. Compared to the unaided condition, the implant provided significant functional gain (mean *M* = 26.1 dB) at 12 months follow-up. Compared to before surgery, average word recognition in quiet at 65 dB and at 80 dB SPL, as well as speech reception threshold in noise, were significantly better at 12 months. However, postoperative air conduction thresholds 6 months after implantation worsened by 10.3 dB (standard deviation SD = 5.8 dB). Postoperatively, three patients had skin problems around the processor, and one of them completely resigned from using the device 5 months after activation. Technical failures occurred in 4 cases. There were 9 out of 10 patients who still used the MET, but only 5 of them used the processor regularly (every day).

**Conclusion:**

Despite changes in the transducer implemented by the manufacturer, we observed a significant number of adverse events in users of the latest generation of MET.

## Introduction

In dealing with sensorineural hearing loss (SNHL), conventional hearing aids (in-the-ear or behind-the-ear) are the first choice [1]. With significant technological progress, acoustic hearing aids (HAs) have developed a range of advantages, but there are still few group of patients who decline to use them or, for some reasons, are unable to use them. Specific areas of dissatisfaction include occlusion of the external ear canal, intolerance of earmolds (pain or itching), and poor sound quality (distortion or feedback) [1, 2].

An alternative option is the semi-implantable active middle ear implant (AMEI). A number of studies have demonstrated that AMEIs can largely eliminate the above-mentioned problems [3–5]. AMEIs have been reported to have better sound quality than conventional HAs [6], and since they bypass the external ear canal, they can be the right solution for patients with chronic external otitis [2]. In clinical practice, some partially implantable devices can be used for the rehabilitation of hearing loss: Vibrant Soundbridge (VSB, Med-El, Insbruck, Austria); Middle Ear Transducer (MET, Otologics LLC, Boulder, CO, U.S.A. and recently Cochlear Ltd., Sydney, NSW, Australia); and Maxum Hearing Implant (Ototronix, Houston, TX, U.S.A.) [7]. In contrast to the VSB implant, which has been used in Poland since 2003 [8–12], the MET system is a relatively new solution.

The first MET implantation in Poland was performed in 2014 by the first author. The MET implant consists of a transmission coil, demodulator, and an actuator mounted into the mastoid with a fixing bracket [13]. The external part contains a magnet, a transmission coil, and an audio processor equipped with microphones that receive the external acoustic signal, which, after processing with a proprietary algorithm, is wirelessly transmitted to the inner part of the system. The indications provided by the manufacturer of the MET are broader than the VSB solution, and it is designed for patients with moderate-to-severe hearing loss [14].

In terms of SNHL, MET devices can be regarded as filling a niche between conventional HAs and cochlear implants. The first published audiological results of MET devices indicated that patients obtained comparable or even better speech understanding than with the best-fitted HAs [5, 15]. However, subsequent scientific papers have reported technical failures and even explantation of the device [1, 2]. To reduce failures, the manufacturer subsequently implemented changes in the design of the transducer. Audiological indications include both SNHL and mixed hearing loss (MHL). To the best of our knowledge, only two papers [13, 16] have scrutinized the safety and efficacy of the latest generation of MET (known as Cochlear MET).

### Objective

The aim of the study was to evaluate the long-term audiological outcomes and safety of the latest generation of middle ear transducer among a group of Polish patients.

## Materials and methods

### Study design

A database consisting of medical records of all patients who had undergone a MET (Cochlear Ltd., Sydney, NSW, Australia) implantation in a tertiary referral ENT centre was carefully examined. CT scans of the patients had been carried out to assess whether implantation of a MET system was possible. The analysis of each patient’s treatment and audiological outcomes was based on full medical documentation. Between October 2014 and December 2018, 12 procedures involving MET implantation were performed. This study was conducted in accordance with the ethical standards of the Institutional Review Board and conformed to the Helsinki declaration. Due to the retrospective nature of the study, no specific informed consent was obtained from the participants.

### Surgery

All patients fulfilled the audiological criteria as recommended by the manufacturer. All procedures were performed in the worse hearing ear by the same senior otosurgeon (H.S.) according to the surgical approach which has been previously described in detail [15]. All patients underwent the same surgical approach and intraoperative complications were not observed. During the activation visit (4–6 weeks after surgery) all patients were fitted with the external Button Audio Processor BAP2.

### Audiometric testing

All patients were given pre and postoperative audiometric assessment consisting of pure-tone audiometry in a soundproof booth using calibrated audiometric earphones. Air conduction (AC) thresholds were assessed at 0.125, 0.25, 0.5, 1, 2, 4, and 8 kHz, and bone conduction (BC) thresholds at 0.25, 0.5, 1, 2, and 4 kHz. Narrow-band noise was used for masking if needed. Hearing threshold measurements were conducted on all patients four times: before implantation of the MET and 6, 12, and 36 months afterwards. The average pure-tone threshold (PTA_4_) for AC and BC were determined at 0.5, 1, 2, and 4 kHz. Means of these frequencies were calculated to give preoperative and postoperative values.

To test the auditory benefits of the implanted device in free-field, the contralateral side was plugged and additionally covered with an over-the-ear phone. A loudspeaker was positioned 1 m in front of the subject. Audiometric tests in free-field were performed at the preoperative period under unaided conditions and at 12 and 36 months postoperatively under aided condition (i.e., with the processor).

The auditory benefits of the MET were evaluated on the basis of free-field hearing thresholds at 0.5, 1, 2, and 4 kHz under unaided and aided conditions. A functional gain (FG) was calculated by determining the difference between the aided and unaided values.

To assess word recognition scores (WRS), the Demenko & Pruszewicz Polish Monosyllabic Word Test in free-field was used. Tests were performed under unaided and aided configurations in quiet at 50, 65, and 80 dB SPL.

Speech reception thresholds (SRTs) in noise were assessed using the Polish Matrix Sentence Test with signal and noise presented from the front. The noise level was fixed at 65 dB SPL and the signal level was changed adaptively. Tests were performed under unaided and aided configurations.

### Statistical analyses

A Shapiro–Wilks test was used to test the assumption of normality. If the assumption of normality was met, a paired samples *t* test was conducted for comparison of preoperative and postoperative results. SPSS IBM v.24 software was used and *p* < 0.05 was considered statistically significant.

## Results

### Study setting and patient selection

Between 2014 and 2018, 11 patients underwent MET implantation, one of whom received bilateral (sequential) implants. Six men and five women had bilateral, slow progressive hearing loss. There were 9 patients with SNHL and two patients with MHL. Two cases with follow-up shorter than 1 year were excluded from the analysis (including one patient who underwent implantation of the other ear). Altogether, 10 patients (10 ears) were included in the study. Age at implantation ranged from 48 to 71 years with a mean of *M* = 58.6 years (SD = 8.4). In most cases, the aetiology of hearing loss could not be determined. In the case of a patient with otosclerosis, incorrect course of the facial nerve canal and a very narrow window niche (< 1.0 mm) were found, significantly reducing the safety of stapedotomy. Preoperatively, 8 out of 10 patients, despite attempts to select appropriate HAs, were dissatisfied and declined to use them. Two patients systematically used the HA before the implantation (in the non-implanted ear) and they continued using them after surgery. Patient information is summarised in Table 1.

**Table 1:** Patient characteristics

Patient	Sex	Age at implantation	Age at diagnosis of hearing loss	Year of implantation	Implant side	Hearing loss type	Etiology of hearing loss	Pre-op hearing aid
1	F	48	28	2014	left	SNHL	unknown	No
2	F	60	15	2014	left	MHL	COM (in childhood)	No
3	F	50	7	2015	right	SNHL	Unknown	Yes
4	M	66	15	2015	right	SNHL	Unknown	Yes
5	F	51	35	2016	right	MHL	Otosclerosis	No
6	M	66	45	2016	left	SNHL	Unknown	No
7	F	62	45	2016	right	SNHL	Unknown	No
8	M	61	39	2016	left	SNHL	Unknown	No
9	M	50	38	2016	left	SNHL	Unknown	No
10	F	72	50	2016	left	SNHL	Unknown	No

*F* female, *M* male, *SNHL* sensorineural hearing loss, *MHL* mixed hearing loss, *COM* chronic otitis media

### Medical and technical complications

Some medical and technical failures associated with the MET were observed, mainly within the first 6 months after implantation. Immediately after surgery, in one case (patient four), dizziness with nausea was observed immediately after surgery; these complaints resolved within 1 month. Patient nine had periodic tinnitus after surgery; currently, he reports a lesser problem with tinnitus severity compared to the short-term postoperative period. In three cases (patients four, seven, and nine), pain complaints and a skin problem around the processor were observed. In two cases, the symptoms did not completely resolve even after a change in magnet strength: one of them (patient four) uses the processor for no more than 3 h per day; the second patient (patient seven) resigned completely from using the device 5 months after activation of the processor. In three cases (patients four, five, and six), technical failures within 6 months of activation meant there was unstable operation of the processor and it generated noise audible to the patients. Similar technical problems with the processor occurred in patient 10 after 2 years of observation. Summarising, 9 of 10 patients were still using the MET after an average observation time of 3.7 years (min 3.2, max 4.9). Five patients were using the processor regularly (every day), while four patients were using the processor irregularly due to a subjective decrease in auditory benefit or a skin problem.

### Pure-tone audiometry

The PTA_4_ for AC and BC thresholds before and after 6, 12, and 36 months from implantation are shown in Table 2. In the short-term follow up (6 months), the average difference between pre and postoperative AC thresholds was 10.3 dB (SD = 5.8) and was statistically significant (*t* = 5.6; *p* < 0.001). In three subjects (patients two, seven, and eight), the AC thresholds shifted upwards by > 10 dB (i.e. a worsening of hearing). At the longer 12- and 36-month follow-up, the analysis of results for nine patients (patient eight was lost to follow-up) showed that the AC thresholds were stable (*t* = 0.4; *p* = 0.703).

**Table 2:** Pure-tone average air and bone conduction before and 6, 12, and 36 months after implantation

Pure-tone average thresholds						
	Air-conduction			Bone-conduction		
Period	*M*	SD	Me	*M*	SD	Me
Preoperative	57.1	10.2	60.0	50.5	20.4	48.7
Result at 6 months	67.4	12.5	66.2	56.0	13.0	54.4
Result at 12 months	71.2	13.5	72.5	58.4	11.2	56.2
Result at 36 months	71.9	15.4	73.7	59.3	10.6	56.2

*M* mean, *SD* standard deviation, *Me* median

The PTA_4_ for BC thresholds was *M* = 50.5 dB (SD = 10.4; Me = 48.8) before surgery and *M* = 56.0 dB (SD = 13.0; Me = 54.4) 6 months after surgery. The difference between them was 5.5 dB (SD = 3.6) and was statistically significant (*t* = 4.8; *p* = 0.001). However, in all cases the BC threshold shift after 6 months was less than ± 10 dB and remained stable at the longer term follow-up.

### Free-field audiometry

Average free-field hearing thresholds for nine patients in unaided conditions was *M* = 72.5 dB HL (SD = 9.8; Me = 76.3) and significantly decreased (*t* = 7.3; *p* < 0.001) after 12 months to *M* = 46.4 dB (SD = 6.7; Me = 47.5). After 36 months it was *M* = 45.3 (SD = 7.1; Me = 46.3) and remained stable in comparison to the 12-month figure (*t* = 0.4; *p* = 0.669).

**Fig1:**
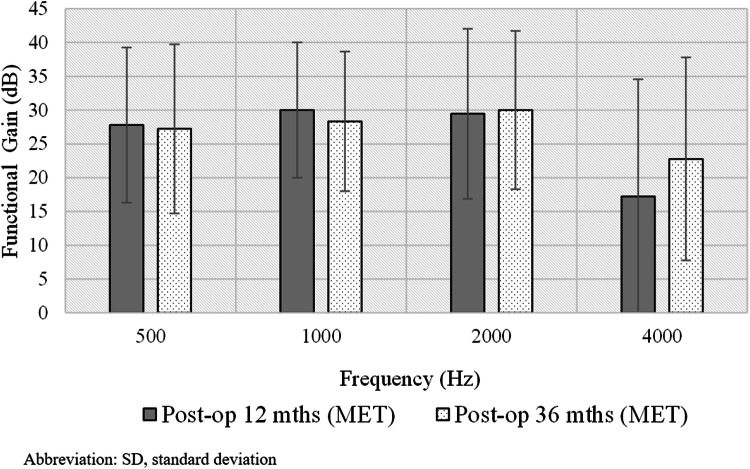
Average functional gain of MET implants for all tested frequencies after 12 and 36 months

Postoperative FG results in MET for all tested frequencies are presented in Fig. 1. The mean FG after 12 months in aided condition compared to the unaided condition was 26.1 dB, and after 36 months it was 27.2 dB and had not changed significantly (*t* = 0.4; *p* = 0.669).

**Fig. 2:**
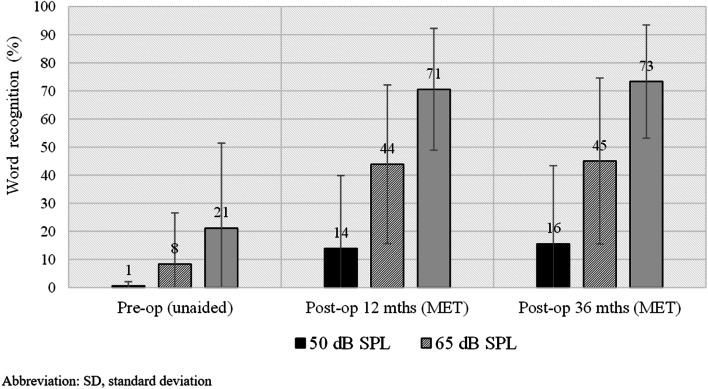
Average word recognition score for unaided and aided configurations in quiet at 50, 65, and 80 dB SPL after 12 and 36 months follow-up

WRS results for nine patients is presented in Fig. 2. At a level of 50 dB SPL, the mean WRS increased insignificantly: first from 6% in unaided conditions to 14% (*t* = 1.64; *p* = 0.139) in aided conditions after 12 months, and then to 16% (*t* = 1.72; *p* = 0.124) after 36 months. At a level of 65 dB SPL, the mean WRS increased significantly (*t* = 4.2; *p* = 0.003) from 8% (unaided) to 44% (aided) after 12 months, and to 45% (*t* = 4.1; *p* = 0.003) after 36 months, resulting in an average improvement of 36% and 37%, respectively. At a level of 80 dB SPL, the mean WRS increased significantly (*t* = 6.0; *p* < 0.001) from 21% (unaided) to 71% (aided) after 12 months and to 73% (*t* = 6.5; *p* < 0.001) after 36 months, resulting in an average improvement of 50% and 52%, respectively.

### Speech understanding in noise

The average SRT in noise improved significantly (*t* = 3.6; *p* = 0.007) from 17.7 dB SNR (SD = 6.4, Me = 21.0) unaided to 8.0 dB SNR (SD = 7.6, Me = 4.6) aided after 12 months. After 36 months it was 7.1 dB SNR (SD = 6.2, Me = 6.0) and did not change significantly (*t* = 0.42; *p* = 0.684) in comparison to 12 months. Thus, SRT in noise resulted in an average improvement of 9.7 dB after 12 months and 10.6 dB after 36 months.

## Discussion

In this article, we have set out our observations of the 2nd generation MET implants. Since the earlier version, these have been modified by the manufacturer to reduce observed complications. In general, the results of our study have shown that the 2nd generation MET implant provided improved auditory function in about half of the operated patients. For the majority of patients, the audiological benefits were adequate but not spectacular. The undoubted advantage of the MET is a broad indication range in terms of dB hearing loss, and it is possible to use this solution even in patients with moderate-to-severe hearing loss who receive limited benefits from conventional acoustical amplification [1, 15, 17].

However, among the 10 patients who underwent surgery, some technical and medical adverse events were noted. Postoperatively, average AC thresholds deteriorated significantly (a shift more than 10 dB) in three patients. In two patients, the shift of hearing thresholds produced poorer auditory benefits, which resulted in irregular use of the device. As a possible cause of the deterioration in AC thresholds, Jenkins and colleagues [15] point to the mass loading of the ossicles by the implant’s transducer. The difficulties and restrictions caused by anatomical limitations during implantation have been described by Kontorinis [17]. Thus, surgeons should be aware of the level of experience and amount of time needed for surgery.

Average BC thresholds also slightly deteriorated, suggesting small intraoperative trauma on the cochlea. However, BC thresholds shifted by no more than 10 dB HL in all cases. In three patients, skin problems were identified, leading in one case to non-usage of the processor. Almost every second patient experienced technical failures. The next cause of complaints concerned periodical tinnitus, which corresponds with similar findings in patients with the Vibrant Soundbridge [9]. Although 9 of 10 implants were still used by the patients, only five of them were used systematically (every day).

Previous publications on the MET device have reported adverse events [13, 16], although most of the events related to the 1st generation implant. Prenzeler et al. [13] investigated implant survival in MET devices for 1st (T1) and 2nd (T2) generation transducers separately over an extended period of time. Unlike the T2, the failure of many T1 devices was found in the first years after implantation. According to the authors, the possible cause is a decrease in coupling efficiency between the implant and the ossicles: in particular, weakness in the ligaments that attach the ossicles to the tympanic walls, slip of the tip of the transducer due to pressure changes in the tympanic cavity or by remodelling of the ossicles caused by chronic pressure, and an increase in stiffness of the ossicular chain. In patients implanted with MET T2, the authors observed no technical failures in an observation period of up to 4 years, although small but significant declines in stimulation efficiency over that period were observed. Similarly, in one of our patients, despite the good preservation of postoperative hearing thresholds, audiological results showed less than expected auditory benefits.

Zwartenko et al. [16]. published results of MET implant survival and postoperative complications. Of the 32 patients with MET (including 4 patients with the 2nd generation MET), the device failure rate was 28%, and 44% devices were replaced or explanted within the first 4 years. All these complications related only to the 1st generation devices. However, our observations of the 2nd generation MET showed that postoperative complications were not uncommon.

In making decisions about qualifying a patient with SNHL for an AMEI, the long-term pros and cons need to be considered. The subjective benefits for patients with SNHL who use an AMEI were reported by Zwartkot et al. [2]. Based on a battery of self-report questionnaires, the authors showed that the majority of patients were content with their AMEI; 71% of patients thought that the AMEI was worth the effort, and 85% reported wearing the device for more than 4 h a day. However, it should be emphasised that in this group, only 8 patients used the MET, which was just 19% of the study participants. In a study by Rameh et al. [18], they demonstrated that improvements in audiological results (free-field FG and SRT) do not reflect real-life patient satisfaction. Over half of patients with semi-implantable Otologics wore a contralateral HA after the implantation. Of these patients, 58% were more satisfied with the behind-the-ear HA compared with the implant. The point to keep in mind is that providing significant functional gain via an implant does not always provide the patient with functional hearing in all acoustic situations, especially in cases of bilateral hearing loss. Moreover, in the case of asymmetric hearing loss, if hearing in the non-implanted ear is at a good level, the patient may as well have high expectations and, as a result, be dissatisfied with the hearing gain offered by the implant. In the current study, we used only audiological test batteries; a limitation of our paper is that the patient’s level of subjective satisfaction in real-life situations was not rated.

In general, previously published experience [13, 16] has been that the medical and technical complications and device failures in 2nd generation MET devices are not uncommon. Those findings are in line with our long-term observations in relation to the latest generation MET. This may explain why 2nd generation MET implants have been withdrawn and are no longer commercially available. An alternative solution proposed by the manufacturer is the fully implantable middle ear implant system, Cochlear Carina [19].

## Conclusion

The audiological indications for the MET implant are broad, which has made it possible to apply this solution to patients with moderate to severe sensorineural or mixed hearing loss. However, despite changes in the transducer implemented by the manufacturer, we have observed a significant number of adverse events in users of the latest generation of MET.

## References

[1] Verhaegen VJO, Mylanus EAM, Cremers CWRJ, Snik AFM (2008). Audiological application criteria for implantable hearing aid devices: a clinical experience at the Nijmegen ORL clinic. Laryngoscope.

[2] Zwartenkot JW, Hashemi J, Cremers CWRJ (2013). Active middle ear implantation for patients with sensorineural hearing loss and external otitis: long-term outcome in patient satisfaction. Otol Neurotol.

[3] Uziel A, Mondain M, Hagen P (2003). Rehabilitation for high-frequency sensorineural hearing impairment in adults with the symphonix vibrant soundbridge: a comparative study. Otol Neurotol.

[4] McRackan TR, Clinkscales WB, Ahlstrom JB (2018). Factors associated with benefit of active middle ear implants compared to conventional hearing aids. Laryngoscope.

[5] Tringali S, Perrot X, Berger P (2010). Otologics middle ear transducer with contralateral conventional hearing aid in severe sensorineural hearing loss: evolution during the first 24 months. Otol Neurotol.

[6] Boeheim K, Pok S-M, Schloegel M, Filzmoser P (2010). Active middle ear implant compared with open-fit hearing aid in sloping high-frequency sensorineural hearing loss. Otol Neurotol.

[7] Kließ MK, Ernst A, Wagner J, Mittmann P (2018). The development of active middle ear implants: a historical perspective and clinical outcomes. Laryngosc Investig Otolaryngol.

[8] Olszewski L, Jedrzejczak WW, Piotrowska A, Skarzynski H (2017). Round window stimulation with the vibrant soundbridge: comparison of direct and indirect coupling. Laryngoscope.

[9] Skarzynski H, Olszewski L, Skarzynski PH (2014). Direct round window stimulation with the Med-El Vibrant Soundbridge: 5 years of experience using a technique without interposed fascia. Eur Arch Oto Rhino Laryngol.

[10] Skarżyński PH, Osińska K, Król B (2018). Use of the Vibrant Soundbridge middle ear implant with short process incus coupler for chronic obstructive inflammation of the external ear canal: case study. J Hear Sci.

[11] Osińska K, Kwasiuk M, Skarżyński PH, Skarżyński H (2018) Vibrant Soundbridge system application in the bilateral congenital malformation of the middle and external ear in the child (Zastosowania systemu Vibrant Soundbridge w obustronnej wadzie wrodzonej ucha środkowego i zewnętrznego u dziecka). Now Audiofonol 7(2):49–58. https://ojs.academicon.pl/na/article/view/2808

[12] Skarżyński H, Porowski M, Mrówka M et al (2015) The Vibrant Soundbridge middle ear implant and the SP coupler in the case of chronic otitis adhesive—case study (Zastosowanie implantu ucha środkowego Vibrant Soundbridge w połączeniu z couplerem typu SP w przypadku przewlekłego zarostowego zapalenia ucha—opis przypadku). Now Audiofonol 4(1):75–78. https://ojs.academicon.pl/na/article/view/2551

[13] Prenzler NK, Kludt E, Giere T (2019). Middle ear transducer: long term stability of the latest generation T2. BioMed Res Int.

[14] Kasic JF, Fredrickson JM (2001). The otologics MET ossicular stimulator. Otolaryngol Clin North Am.

[15] Jenkins HA, Niparko JK, Slattery WH (2004). Otologics middle ear transducer^TM^ ossicular stimulator: performance results with varying degrees of sensorineural hearing loss. Acta Otolaryngol (Stockh).

[16] Zwartenkot JW, Mulder JJS, Snik AFM (2016). Active middle ear implantation: long-term medical and technical follow-up, implant survival, and complications. Otol Neurotol.

[17] Kontorinis G, Lenarz T, Schwab B (2010). Anatomic limitations in implantation of middle ear transducer and carina middle ear implants. Laryngoscope.

[18] Rameh C, Meller R, Lavieille J-P (2020). Long-term patient satisfaction with different middle ear hearing implants in sensorineural hearing loss. Otol Neurotol.

[19] da Peixoto M, C, Miranda C, Bento M, et al (2019) The first results of a totally implanted active middle ear device. Eur Arch Otorhinolaryngol. 10.1007/s00405-019-05557-8 10.1007/s00405-019-05557-8 31342145

